# Molecular Regulation of the Polycomb Repressive-Deubiquitinase

**DOI:** 10.3390/ijms21217837

**Published:** 2020-10-22

**Authors:** Cameron J. Reddington, Matthias Fellner, Abigail E. Burgess, Peter D. Mace

**Affiliations:** Department of Biochemistry, School of Biomedical Sciences, University of Otago, P.O. Box 56, 710 Cumberland St., Dunedin 9054, New Zealand; redca227@student.otago.ac.nz (C.J.R.); matthias.fellner@otago.ac.nz (M.F.); abigail.burgess@otago.ac.nz (A.E.B.)

**Keywords:** H2AK119Ub, Polycomb Repressive-Deubiquitinase, PR-DUB

## Abstract

Post-translational modification of histone proteins plays a major role in histone–DNA packaging and ultimately gene expression. Attachment of ubiquitin to the C-terminal tail of histone H2A (H2AK119Ub in mammals) is particularly relevant to the repression of gene transcription, and is removed by the Polycomb Repressive-Deubiquitinase (PR-DUB) complex. Here, we outline recent advances in the understanding of PR-DUB regulation, which have come through structural studies of the *Drosophila melanogaster* PR-DUB, biochemical investigation of the human PR-DUB, and functional studies of proteins that associate with the PR-DUB. In humans, mutations in components of the PR-DUB frequently give rise to malignant mesothelioma, melanomas, and renal cell carcinoma, and increase disease risk from carcinogens. Diverse mechanisms may underlie disruption of the PR-DUB across this spectrum of disease. Comparing and contrasting the PR-DUB in mammals and *Drosophila* reiterates the importance of H2AK119Ub through evolution, provides clues as to how the PR-DUB is dysregulated in disease, and may enable new treatment approaches in cancers where the PR-DUB is disrupted.

## 1. Introduction

Cells face the fundamental challenge of storing a vast genome in limited three-dimensional space, while allowing for orderly access to DNA. Eukaryotic cells meet this challenge by wrapping DNA around histone proteins to form nucleosomes and by efficiently packing nucleosomes together into chromatin. Changes to DNA accessibility and nucleosome packaging are largely governed by histone modifications, which alter DNA accessibility and transcription. Amongst the raft of possible post-translational modifications, histone C-termini are frequently modified by attachment of a protein measuring 76 amino acids long called ubiquitin [1]. Monoubiquitination of histones H2A and H2B differentially influence nucleosomal packaging [2]—H2BK120Ub is associated with actively transcribed chromatin, while H2AK119Ub is associated with repression of developmental genes [1]. Initially, H2AUb (H2AK119Ub in mammals, H2AK118Ub in *Drosophila*) was thought to have a role in the maintenance of Polycomb Group (PcG)-mediated gene repression in *Drosophila* [3]. However, H2AK119Ub is now considered essential for mammalian PcG protein localisation and gene repression [4].

Ubiquitination occurs in a multi-step process, with ubiquitin chains successively built through the activities of ubiquitin-activating (E1), conjugating (E2) and ligase (E3) proteins. Conversely, ubiquitin is removed by deubiquitinase enzymes [5]. The incidence of oncogenic mutations in the genes encoding components of the protein complexes that write and erase ubiquitin histone marks has focused interest on their regulation and on how the process as a whole is integrated with other histone modifications.

Broadly speaking, PRC1 and PRC2 catalyse the modification of histones through the ubiquitination of H2AK119 and tri-methylation of H3K27, respectively. The activities of Polycomb complexes 1 and 2 (PRC1 and 2) are discussed in depth elsewhere in this issue, and have been researched extensively in the 70 years since their discovery. This review will highlight our understanding of H2AK119Ub regulation, with a particular focus on the protein complex capable of removing H2AUb, the Polycomb Repressive-Deubiquitinase (PR-DUB) complex, which has come to light only in the past decade.

## 2. PR-DUB, the Polycomb-Repressive Deubiquitinase

**Figure 1:**
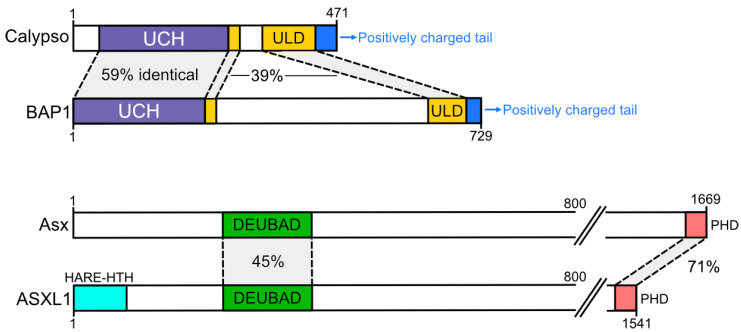
Domain architectures of the human and *Drosophila* Polycomb Repressive-Deubiquitinase (PR-DUB) components. Sequence identity between conserved domains is shown. Note that the scale is broken at 800 amino acids and continued at 1500. Figure adapted from [8].

Two proteins in *Drosophila*, Calypso and Additional sex combs (Asx), form the PR-DUB complex. Calypso is the catalytic deubiquitinase within the complex, which hydrolyses H2AUb when activated by Asx [6] (Figure 1). In mammals, a single homolog of Calypso exists (BRCA1-associated protein 1; BAP1), which can be activated by one of three Asx-like (ASXL) proteins (ASXL1–3). BAP1, ASXL1, and ASXL2 are expressed across a range of cell types; ASXL3 has a restricted expression pattern and is enriched in the brain and in pluripotent respiratory epithelial cells [7].

Calypso and BAP1 contain two highly conserved regulatory domains—the ubiquitin C-terminal hydrolase (UCH) and UCH L5-like domain (ULD) [9]. The UCH conveys the deubiquitinase activities of the PR-DUB, while the ULD interacts with the deubiquitinase-associated domain (DEUBAD) of Asx or ASXL1–3. Association of the ULD and DEUBAD domains increases the affinity of the PR-DUB for ubiquitin and is required for deubiquitination [6,9,10]. At the C-terminus of their ULD domains, Calypso and BAP1 each have positively charged C-terminal extensions, which are required for binding the net negatively charged nucleosome [8,9]. A major difference in domain architecture between BAP1 and Calypso is an insertion of around 380 amino acids into the ULD domain of BAP1, which is proposed to enable a broader range of interactions by the mammalian protein [11,12,13] (Figure 1).

The DEUBAD domains of Asx and ASXL1–3 are essential for interaction with and activation of Calypso and BAP1. In addition, ASXL1–3 and Asx all contain a predicted atypical plant homeodomain (PHD) at their C-terminus—PHD domains conventionally bind specific histone methylation marks, which could potentially overlap with H2AK119Ub. Mammalian ASXL proteins differ from their *Drosophila* counterpart in that ASXL1–3 each possess a DNA binding HB1, ASXL, and restriction endonuclease helix-turn-helix (HARE-HTH) domain at their N-terminus, which is noticeably absent from Asx [14] (Figure 1).

## 3. Regulation of PR-DUB Catalytic Activity

The N-terminal UCH domain of BAP1 belongs to a family of cysteine proteinases, which are found in all kingdoms of life and are characterised by a catalytic cysteine–histidine–aspartate triad. Beyond the core catalytic residues, UCH deubiquitinases share a common feature adjacent to the catalytic site—the active-site crossover loop. This crossover loop is thought to discriminate between substrates on the basis of size [15,16,17]. Some UCH family members have small, restrictive crossover loops and cleave ubiquitin from small peptides. Others have much larger crossover loops, allowing for the cleavage of ubiquitin from larger substrates. BAP1 has the longest and most permissive crossover loop amongst the UCH hydrolases [17]. BAP1’s elongated crossover loop enables it to cleave ubiquitin from protein substrates, including itself, Asx-like proteins, host cell factor 1 (HCF-1), and of course H2AK119Ub [12,18].

Of the five UCH deubiquitinase proteins in humans, the closest structural homolog of BAP1 or Calypso is ubiquitin carboxyl-terminal hydrolase isozyme L5, UCH-L5; also known as UCH37, which also contains a C-terminal ULD. UCHL5 carries out deubiquitination at the 26S proteasome [19,20,21,22] when activated by the proteasomal DEUBAD-containing protein Rpn13. Because of their shared domain architecture, our current understanding of PR-DUB catalytic activity is built upon both recent crystal structures of the *Drosophila* PR-DUB complex and prior structures of the UCHL5–Rpn13 complex.

**Figure 2:**
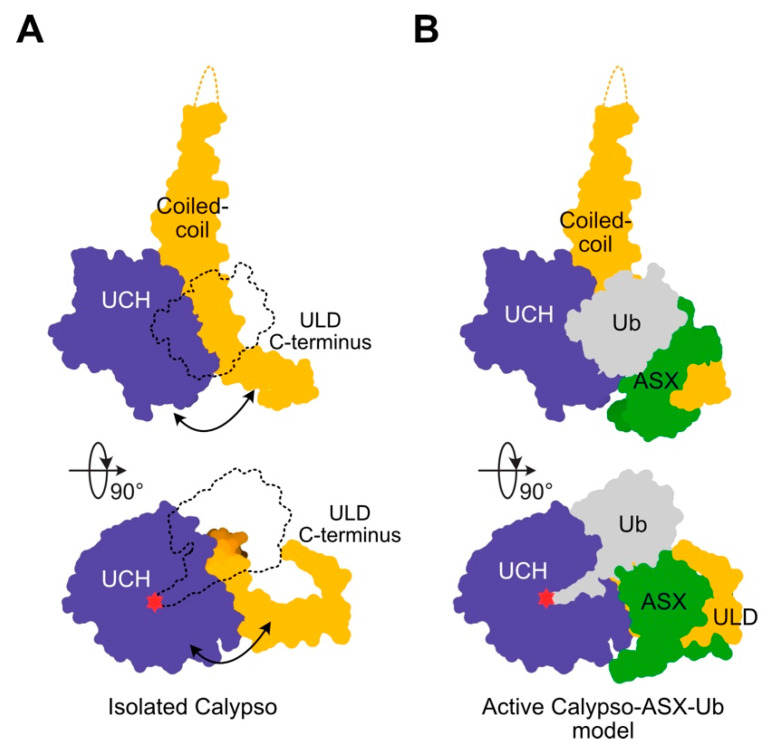
Crystal structure of the *Drosophila* PR-DUB complex. The catalytic site is noted in red, while other components of the PR-DUB are coloured as in Figure 1. (**A**) Structure of the UCH and ULD domains of Calypso. The likely binding site of ubiquitin is shown in silhouette, while Asx is omitted to illustrate that potential flexibility in the C-terminus of the ULD would interfere with the ubiquitin binding site (arrow). (**B**) Structure of the Calypso-Asx heterodimer; ubiquitin binding site modelled on UCH-L5-Rpn13-Ub structure (PDB: 4UEL). Note the shared contributions of Calypso and Asx in forming the ubiquitin binding pocket.

Two near-identical structures of Calypso bound to the DEUBAD of Asx were recently solved and provide structural insight into deubiquitination by the PR-DUB [8,23]. In these structures, the UCH domain of Calypso is linked to the ULD through a coiled-coil hairpin. When bound to the DEUBAD of Asx, the C-terminal portion of the ULD is trapped in a conformation protruding away from the UCH active site to allow for deubiquitinase activity. Both of the crystal structures of Calypso and Asx are similar to UCH-L5 complexed with the DEUBAD of its proteasomal activator, regulatory particle subunit 13 (Rpn13) [24,25]. Structures of UCH-L5-Rpn13 bound to ubiquitin are available. Using these structures to superimpose ubiquitin into the PR-DUB reveals that when in complex, Calypso and Asx act together to form a composite ubiquitin binding site [10,24,25] (Figure 2). Notably, UCH-L5 is capable of binding two interchangeable DEUBAD domains from Rpn13 or INO80G, which lock UCHL5 in either active or inactive states, respectively. The DEUBAD of the Asx-like proteins perform an exclusively activating function and lock BAP1 in an active state.

## 4. Higher Order Complex Formation by the PR-DUB

The tight 1:1 interaction between deubiquitinase (BAP1 or Calypso) and Asx-like protein is well established to be crucial for PR-DUB activity [26,27]. However, in Calypso and BAP1, a higher oligomeric state also plays a role. The recent Calypso-Asx crystal structures were solved in two independent spacegroups, but share the same packing arrangement around the coiled-coil hairpins. This arrangement allows the coiled-coil hairpin of Calypso to form an extended dimer between two Calypso molecules [24,28]. Biophysical analyses show that the 2:2 bidentate oligomer forms in solution and is concentration-dependent, so is likely to only occur in scenarios where Calypso is enriched at higher concentrations. Disruption of the coiled-coil interface does not affect the inherent catalytic activities of Calypso-Asx or BAP1-ASXL1, but impairs Calypso or BAP1 recruitment to nucleosomes [8].

**Figure 3:**
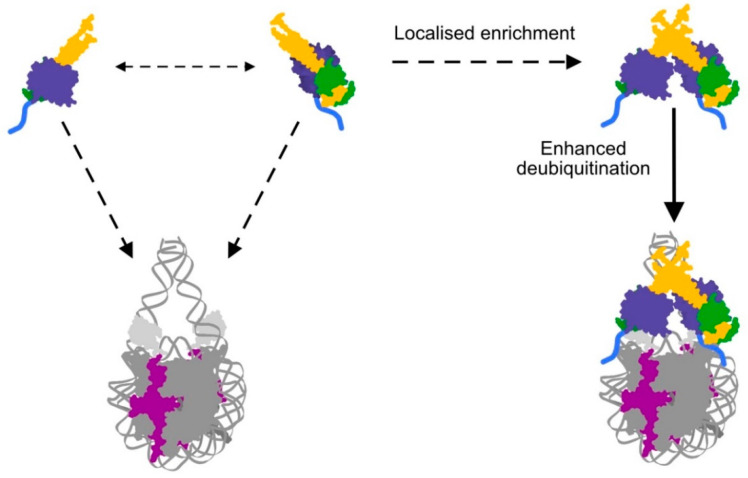
Model of PR-DUB localisation proposed by Foglizzo et al. [8]. A nucleosome is shown in dark grey, with histone H2A highlighted in magenta and ubiquitin attached (light grey). The Calypso-Asx dimer has poor affinity for nucleosomes (dashed arrow). Formation of a bidentate PR-DUB containing 2 Asx and 2 Calypso proteins facilitates efficient binding of the ubiquitinated nucleosome (solid arrow) through the positively charged Calypso C-terminal tail (blue). Localised enrichment of the PR-DUB to H2AK119Ub enables heterodimerisation and reinforces an integral role for nucleosomal localisation in PR-DUB activity.

Bringing together two key observations, namely that the C-terminal positively charged tail is required for nucleosome recruitment and that the 2:2 bidentate oligomer has increased activity on the H2AK119Ub nucleosome substrate, allowed Foglizzo et al. to propose a model for PR-DUB nucleosome recruitment and activity [10] (Figure 3). In this model, PR-DUB dimerisation increases binding avidity for the nucleosome through the positively charged C-terminal tail and subsequently enhances catalytic activity. The concentration dependence of the 2:2 complex formation means that maximal activity is likely to occur only when the PR-DUB is locally enriched, meaning enrichment of the complex to specific regions of chromatin is critical. Thus, mechanisms that localise the PR-DUB in healthy cells, and conversely mechanisms by which PR-DUB targeting is disrupted in disease, are highly relevant to understanding H2AK119Ub regulation and gene expression.

## 5. PR-DUB Localisation and Recognition of H2AK119Ub

A full understanding of how PR-DUB activity is directed is still being developed, but it is likely to include a combination of interactions mediated by BAP1/Calypso and Asx-like proteins with histone marks, specific DNA sequences, and co-recruitment to related histone-modifying complexes. The importance of H2AK119Ub in Polycomb recruitment has been debated since the discovery of Polycomb-repressive systems in *Drosophila*. However, recent work highlights that H2AK119Ub is central to mammalian Polycomb recruitment and transcriptional repression. These studies reinforce the importance of the complexes that regulate H2AK119Ub, specifically PRC1 and the PR-DUB [29,30,31]. Since PRC1 attaches and PR-DUB detaches H2AK119Ub, localisation of the two complexes could logically be expected to be coordinated. Notably, PRC1 localisation is a complicated process that differs drastically between species. In a similar vein, localisation of the PR-DUB may differ between species. Here, we will outline mechanisms that may regulate localisation of the PR-DUB in both *Drosophila* and mammals, particularly in the context of recent studies of PRC1 targeting and H2AK119Ub deposition.

### 5.1. Relative Arrangement of PRC1 and PRC2

**Figure 4:**
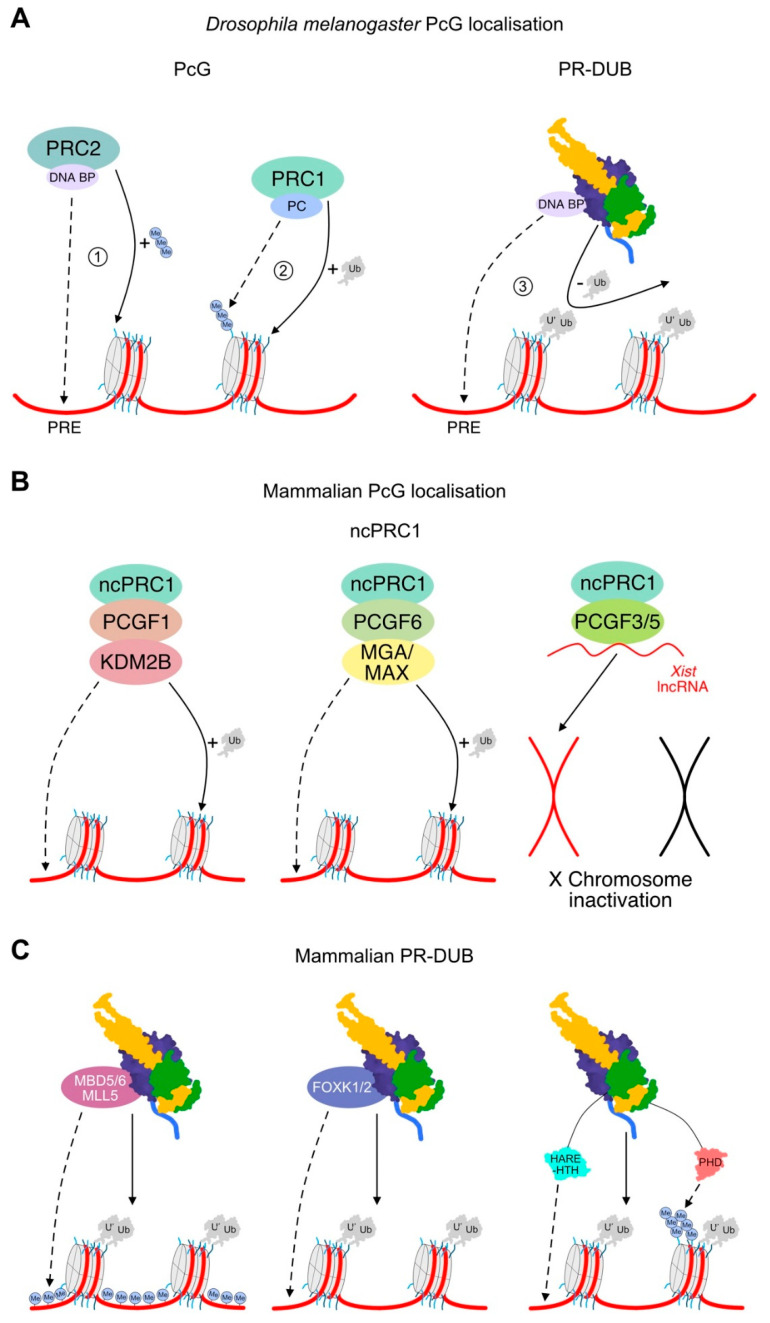
Model of PcG and PR-DUB localisation. (**A**) *Drosophila melanogaster* PcG localisation. (1) PRC2 interacts with DNA binding proteins (DNABP) to recruit PRC2 to Polycomb Response Elements (PREs). PRC2 then deposits H3K27me3. (2) PRC1 recognises H3K27me3 through Polycomb (PC), oligomerises to compact chromatin, and minimally ubiquitinates H2AK118Ub (K118 is modified in *Drosophila*). (3) It is likely that the *Drosophila* PR-DUB would recognise PREs through interacting with DNABPs, removing H2AUb, and facilitating transcriptional activation. (**B**) Mechanisms of mammalian ncPRC1 localisation: ncPRC1-PCGF1 interacts with Lysine Demethylase 2B (KDM2B) to recognise non-methylated DNA; ncPRC1-PCGF6 interacts with DNA binding proteins, including MGA-MAX; ncPRC1-PCGF3/5 interact with the *Xist* lncRNA and repress X chromosome expression. (**C**) Potential mechanism of mammalian PR-DUB localisation. Methyl binding domains 5 and 6 (MBD5/6) and mixed lineage leukaemia 5 (MLL5) appear likely to specify recruitment to methylated CpG islands, and FOXK1/2 appear likely to specific DNA sequences. The PHD and HARE-HTH domains of ASXL1–3 appear likely to mediate recruitment to histone methylation and DNA sequences, respectively.

When considering PR-DUB localisation, it is worth reiterating the mechanisms understood to localise PRC1, given that the PR-DUB antagonises the H2AK119Ub mark generated by the PRC1 complex. In *Drosophila*, PRC1 localisation is the result of a hierarchical system stemming from association of PRC2 with DNA binding factors, including Pleohomeotic and Pleohomeotic-like factors. These DNA binding proteins recruit PRC2 to conserved cis-regulatory motifs, named “Polycomb response elements” [32]. Recruitment of PRC2 to a Polycomb response element initiates H3K27me3 deposition [33]. PRC1 is recruited to H3K27me3 through the chromodomain of Polycomb, a core component of PRC1, hence recruiting PRC1 to genes earmarked for repression [3]. Oligomerisation of chromatin-bound PRC1 drives nucleosomal compaction and long-range interactions between distant Polycomb target sites [34] (Figure 4A).

In contrast, mammalian PRC1 has diverged from *Drosophila*—two Sex Combs Extra homologs, really interesting new gene (RING) 1A and 1B, interchangeably associate with Polycomb group factors to form six possible PRC1 complexes. Six different Polycomb group RING finger proteins (PCGFs) may be incorporated into PRC1, forming either a canonical (cPRC1) or non-canonical PRC1 (ncPRC1) complex [35]. The cPRC1 complexes (assembled around PCGF2/4) mimic *Drosophila* PRC1 and oligomerise to repress transcription. However, conditional removal of cPRC1 and PRC2 in mammalian cells causes relatively few gene expression defects, suggesting that the contributions of cPRC1 compaction to mammalian gene repression are minimal [29,30].

Mammalian ncPRC1 complexes each have unique subsets of interacting partners to convey different modes of localisation [29,30]. For instance, human PcG proteins are commonly bound at CpG islands. Cytosine methylation represses mammalian gene transcription, meaning unmodified CpG islands correlate to open, actively expressed chromatin and are a viable mechanism for targeting the transcriptionally repressive PcG proteins [33]. PCGF1-containing ncPRC1 complexes are localised to non-methylated CpGs through lysine demethylase 2B, which binds non-methylated CpG islands through its ZF-CxxC DNA binding domain [36]. Alternatively, PCGF3 and PCGF5 form nearly identical protein complexes [37], interacting with the *Xist* lncRNA to implicate them in *Xist*-mediated X chromosome inactivation [30]. On the other hand, PCGF6 forms complexes with a range of interchangeable DNA binding proteins, including MGA/MAX or E2F6, allowing nucleosomal recruitment to occur in a cell-type- and context-specific manner, depending on the incorporated DNA binding proteins [38] (Figure 4B).

### 5.2. PR-DUB Localisation through Interaction Partners

Intriguingly, proteins associated with the mammalian PR-DUB appear tailored toward the recognition of elements that would logically promote deposition of H2AUb-specific DNA sequence motifs and the CpG island methylation state. Parallel to the regulation of PRC1 between *Drosophila* and mammals, there is likely evolutionary divergence in localisation of the PR-DUB, with a minimal interactome of the PR-DUB in *Drosophila* and more complex mechanisms at play in mammals.

Because relatively little is known about the interacting partners of the *Drosophila* PR-DUB, we can only speculate that the primary mechanism of targeting Calypso activity is through its activating partner Asx. Notably, the primary sequence of Asx differs substantially from ASXL1–3; other than the conserved DEUBAD and PHD domains, Asx lacks an N-terminal HARE-HTH and contains several stretches of poly-glutamines and poly-alanines, which are absent from ASXL1–3. Such poly-glutamine and -alanine stretches are common in *Drosophila* proteins, which bind DNA binding factors, and while uncharacterised in Asx may bind proteins capable of recruiting the *Drosophila* PR-DUB to Polycomb response elements [39]. Intriguingly, BAP1 is also known to interact with Yin Yang 1, the human homolog of Pleohomeotic [13]. Yin Yang 1 is unlikely to mediate PR-DUB localisation in mammals, as Yin Yang 1 is not thought to have a role in mammalian PcG protein recruitment [33]. Instead, association of Yin Yang 1 with BAP1 could be the remnant of an ancient association between Pleohomeotic and Calypso, which would have allowed for the recognition of Polycomb response elements by the *Drosophila* PR-DUB complex.

Proteomic and functional studies have established a range of proteins as interactors of the PR-DUB in mammals. Core BAP1 interactors include forkhead box K1 and K2 (FOXK1/K2), methyl-CpG binding domains 5 and 6, and host cell factor 1 [6,40]. FOXK1 and K2 are DNA binding transcription factors, which may convey sequence-specific binding of BAP1 and detect H2AUb deposited by PCGF6-PRC1 [41,42,43]. Methyl-CpG binding domains 5 and 6 contain methyl-CpG binding domains and bind BAP1 in a mutually exclusive fashion. Based on their names, methyl-CpG binding domains 5 and 6 would be expected to bind methylated CpG islands and detect H2AUb deposited by PCGF1-PRC1. However, a role for these proteins in methyl-CpG recognition has not yet been identified [44]. Instead, transient BAP1 interactors, including mixed lineage leukaemia 5, are bound at richly methylated regions of DNA and likely contribute to enrichment of PR-DUB at these sites [40,45]. BAP1 also interacts with host cell factor 1, a transcriptional coactivator capable of recruiting a variety of transcription factors and proteins capable of ensuring downstream transcriptional activation [13,40]. Of particular interest, O-linked GlcNAc-transferase 1 associates with the PR-DUB through an interaction with host cell factor 1 [46]. O-linked GlcNAc-transferase-1-mediated GlcNAcylation of histone H2B can promote H2BK120 ubiquitination, increasing overall levels of transcription [47]. Thus, assembly of O-linked GlcNAc-transferase 1 into the PR-DUB complex may promote transcriptional activation upon removal of H2AK119Ub.

As well as activating PR-DUB deubiquitination, the domains of ASXL1–3 appear to coordinate aspects of targeting the PR-DUB to chromatin. For instance, the C-terminal PHD is hypothesised to bind histone modifications, with strong overlap with H2AK119Ub [14,48]. Meanwhile, the ASXL HARE-HTH, a putative DNA binding domain, is thought to detect mammal-specific features of Polycomb localisation, either in binding to derivatives of methylated DNA or to DNA sequence motifs [14]. Strikingly, the ASXL HARE-HTH is absent from Asx, reinforcing a mammal-specific role for this domain in recognising the deposition of H2AK119Ub (Figure 4C).

Overall, PR-DUB interactors appear to recognise elements that drive PRC1 localisation; in mammals this involves CpG island methylation and DNA sequence motifs, while in *Drosophila* this involves Polycomb response elements (Figure 4). In healthy cells, these interacting proteins likely help to establish the localised enrichment and heterodimerisation of the PR-DUB complex, conveying downstream deubiquitination of H2AK119Ub and subsequent transcriptional activation. Notably, some studies suggest a role for PR-DUB in transcriptional repression, centred around a proposed interaction of the ASXL proteins with components of the PRC2 complex [49,50]. Association of ASXL proteins with PRC2 is yet to be borne out in proteomic studies [40,51], so further investigation of how the PR-DUB contributes to transcriptional repression is an area for future investigation. Moreover, in 2019 it was demonstrated that PR-DUB plays a key role in protecting actively transcribed genes from silencing by directly limiting the ability of PRC1 to ubiquitinate and silence gene expression [52]. Thus, it appears that the PR-DUB can contribute to activation, repression, and maintenance of transcription, and further mechanistic studies of each scenario are required to enhance understanding of PR-DUB function in both health and disease.

## 6. PR-DUB and Human Disease

### 6.1. Consequences of PR-DUB Disruption

Mutations that affect the components of the PR-DUB complex can lead to global changes in the chromatin status, and therefore the transcriptional environment of the cell. As such, mutations in the PR-DUB are overrepresented in a number of cancers. *BAP1* mutations are often described as cancer drivers, most notably in the case of BAP1 tumour predisposition syndrome (BAP1-TPDS; [48]). BAP1-TPDS first came to light through studies of familial clusters with increased rates of malignant mesothelioma despite minimal exposure to asbestos. Mesothelioma is highly aggressive and hard to treat, but is normally tightly correlated to asbestos exposure. Analysis of BAP1-TPDS patients has revealed an association between mesothelioma development and *BAP1* mutations. In BAP1-TPDS, inactivating germline *BAP1* mutations cause development of mesothelioma, melanoma, and other neoplasms [53,54,55]. BAP1-TPDS is highly penetrant, with ~85% of heterozygous carriers diagnosed with cancer [53]. Somatic mutations in *BAP1* occur most frequently in pleural and eye cancers (21% and 32%, respectively; as annotated in the Catalogue of Somatic Mutations in Cancer (COSMIC) database [56]). Moreover, *BAP1* mutations occur in ~15% of clear cell renal cell carcinomas (CCRCC), and patients bearing such mutations have a particularly poor prognosis relative to other common molecular subtypes of CCRCC [57].

*BAP1* mutants exhibit haploinsufficiency, where a single functional copy of *BAP1* does not protect from the effects of asbestos exposure [58]. However, knock-in mice studies show that the catalytic inactivation of BAP1 leads to Caspase-3 cleavage, a hallmark of apoptosis [59]. This presents an unexpected juxtaposition, where inactivating mutations in the same protein can activate an apoptotic pathway through Caspase-3, but can also induce tumorigenesis in patients. A CRISPR-Cas9 screen has revealed a key mechanism linking BAP1 loss and RING1B-mediated H2AK119Ub deposition, which goes a long way towards explaining this juxtaposition [59]. When BAP1 is depleted, RING1B can repress the transcription of *Bcl2* and *Mcl1*, two anti-apoptotic genes that prevent apoptosis when appropriately expressed. Thus, loss of BAP1 activity prevents *Bcl2* and *Mcl1* expression and triggers apoptosis. The pro-apoptotic role of BAP1 is cell-type specific, as loss of BAP1 expression does not induce apoptosis in melanocytes or in mesothelial cells—RING1B does not coordinate the expression of *Bcl2* or Mcl1 in these tissues. Instead, BAP1 loss leads to cellular differentiation, proliferation, or both [59].

In contrast to the aetiology of cancers associated with mutations in *BAP1*, mutations in *ASXL1* and *ASXL2* are strongly linked to myeloid cell cancers [49,60,61,62,63], with ~11% of haematopoietic cancers bearing *ASXL1* mutations (COSMIC). As with *BAP1*, both *ASXL1* and *ASXL2* are haploinsufficient tumour suppressors, where mutations can drive pathology as heterozygous mutants alone or in combination with secondary mutations [49,60,63]. Somatic *ASXL1* mutations are found in various myeloid and myelomonocytic leukaemias, and are always associated with poor prognosis [64,65,66]. However, there is much debate in the literature as to whether truncating *ASXL1* mutations represent a loss-of-function or gain-of-function [49,51,61,67]. Beyond cancer, *de novo* truncating mutations in *ASXL1*, *ASXL2*, and *ASXL3* have separately been identified in Bohring–Opitz, Bainbridge–Ropers, and related syndromes [68,69,70]. The presence of similar mutations in both cancer and developmental syndromes highlights the important role of ASX-like proteins in initially determining, and then maintaining, appropriate developmental gene expression in humans.

### 6.2. Mechanisms of PR-DUB Disruption through Mutation

Structures of the *Drosophila* PR-DUB complex have allowed cancer associated mutations to be mapped to the equivalent residues of the complex [8,23]. Cancer-derived mutations are enriched in functionally important regions of BAP1—within the catalytic domain of BAP1, disrupting the catalytic triad or structural integrity of the domain; and within the ubiquitin binding site or crossover loop, disrupting the binding interface between BAP1 and ASXL1 [8,23]. Missense mutations also occur in *ASXL1/2*, which would impair PR-DUB catalytic activity. Specifically, mutations in the conserved NEF motif within the DEUBAD domain of ASXL1/2 occur [9]. NEF mutations do not impair binding of ASXL1 to BAP1, but rather impair the activity of BAP1. This NEF site sits near the ubiquitin binding pocket of BAP1 and is important for the stabilisation of ubiquitin within this pocket, and therefore its removal [8,23].

In addition to missense mutations disrupting catalytic activity, nonsense mutations that truncate the BAP1 protein are also recurrent in sequenced patient tumours. Depending on the severity of the truncation, this can either result in a protein that lacks the ASXL1 docking site in the ULD or lacks the positively charged C-terminal extension. Loss of the ASXL1 docking site results in a protein that has impaired activity and is unable to remove ubiquitin from nucleosomes. The C-terminal extension is not required for ASXL1 binding [9]. Mutations that result in a loss of the C-terminal extension would have impaired ability to bind nucleosomes, but retain intrinsic activity on a minimal substrate. These lose their nuclear localisation, showing the importance of this region for proper targeting [9,71]

Most cancer-associated *ASXL1* mutations enhance the catalytic function of BAP1, and therefore decrease levels of H2AK119Ub. The majority of *ASXL1* mutations occur in the last exon, exon 12, and result in a truncated protein that lacks the C-terminus, including the putative PHD domain [72]. It is predicted that this could mean that the mutant protein escapes nonsense-mediated decay [73]. Moreover, in mice and embryonic stem cell models, these frameshift mutations, when introduced, mirror disease progression, often only requiring a heterozygous mutation, and have high penetrance [11,64,74,75].

If the ASXL PHD can recognise certain histone modifications, as previously suggested [14], truncation of *ASXL1–3* would lead to mislocalisation of the PR-DUB. Aberrant targeting of the PR-DUB complex would then lead to the non-specific removal of H2AK119Ub, and eventually global erasure. However, truncation of ASXL proteins has been shown to enhance monoubiquitination of a site within the DEUBAD of ASXL [64,76]. DEUBAD monoubiquitination is known to stimulate BAP1 deubiquitinase activity, arguing for a role of the ASXL PHD in regulating ASXL ubiquitination [64]. These findings were reinforced by Daou et al., who proposed a model wherein full-length ASXL is monoubiquitinated when in complex with BAP1. In the model, an inactive PR-DUB is unable to auto-deubiquitinate, is polyubiquitinated, and becomes targeted for proteasomal degradation. However, truncated versions of ASXL1–3 are aberrantly protected from polyubiquitination and are constitutively monoubiquitinated, leading to enhanced BAP1 deubiquitinase activity and widespread removal of H2AK119Ub [76]. It remains possible that loss of the PHD domain can trigger both mechanisms—loss of specific chromatin localisation and aberrant ASXL ubiquitin-based regulation. Because truncating mutations are the most common form of oncogenic ASXL mutation, the mechanisms at play certainly warrant further research.

Further reports have shown that ASXL truncations can affect the PR-DUB interactome by disrupting some core interactions and promoting others. For instance, a recent study showed that *ASXL* truncations cause decreased interactions with FOXK1/2 [77]. As described above, FOXK1/2 are sequence-specific transcription factors, so disrupted interactions would be expected to cause mislocalisation of the PR-DUB. *ASXL1* truncations may also induce novel interactions. Recent work suggests that specific ASXL1 truncating mutations, but not wild-type ASXL1, can bind the Bromodomain-containing protein 4 (BRD4) [51]. BRD4 is a protein that is able to bind transcriptionally activating histone acetylations, including H3K9ac [78]. An acquired interaction with BRD4 would likely cause mislocalisation of the PR-DUB complex and aberrantly activate transcription [51]. A putative site of BRD4–ASXL interaction has been identified within full-length ASXL3 [79], although the authors report no interaction between BRD4 and ASXL1 or 2. Furthermore, equivalent ASXL truncations have been shown to be sensitive to HDAC inhibitors, targeting against BRD4 activity [80], offering hope that an improved understanding of mechanisms underlying disrupted PR-DUB regulation can lead to improved options for the treatment of cancers where these disruptions occur.

## 7. Conclusions

The activity of the PR-DUB deubiquitinase is regulated at multiple levels, namely by assembly of a tight complex between BAP1 or Calypso and an Asx-like protein, a 2:2 “double-heterodimer” complex likely to only occur upon localised enrichment in specific regions of chromatin [8], and interactions with a range of cellular binding partners likely to modulate specificity and function. An emerging scenario is that disruption of any of these steps has the potential to impair PR-DUB function and change the cellular epigenetic landscape. While significant progress has been made since discovery of the PR-DUB complex, a more comprehensive molecular model of PR-DUB regulation is crucial to understanding epigenetic protection against cancer. Furthermore, BAP1 mutation in cancer cells predicts mortality and recurrence across various cancer types, including colorectal, renal, uveal melanoma, and lung adenocarcinoma, but is less predictive in others [81,82,83]. This supports the idea that BAP1 mutations do not dictate disease progression alone, however the wider network into which the PR-DUB is integrated is crucial. Continued interrogation of this system is essential to fully understand the importance of PR-DUB function in healthy epigenetic maintenance, disease risk, and the potential for targeted therapeutic strategies.
